# How Domain Segregation in Ionic Liquids Stabilizes Nanoparticles and Establishes Long-Range Ordering—A Computational Study

**DOI:** 10.1021/acsnano.4c04581

**Published:** 2024-07-27

**Authors:** Kalil Bernardino

**Affiliations:** Laboratório de Química Computacional, Departamento de Química, Universidade Federal de São Carlos, Rod. Washington Luiz S/N, 13565-905 São Carlos, Brazil

**Keywords:** nanoparticle aggregation, nanoparticle stabilization, ionic liquids, potential of mean force, molecular dynamics simulations

## Abstract

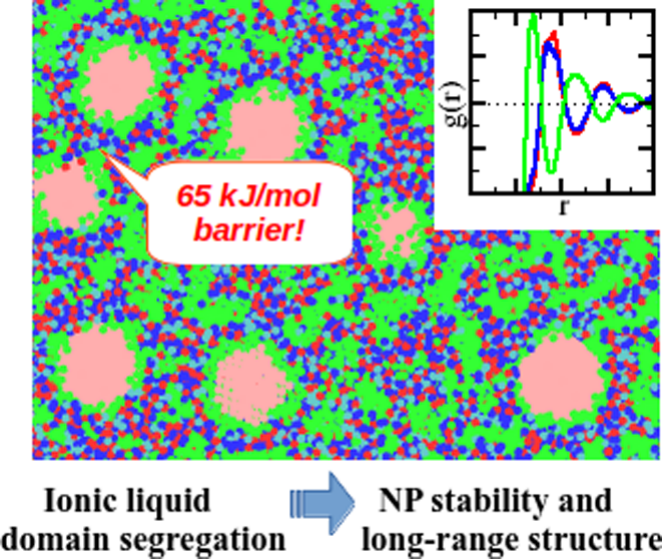

Due to their physical properties including high thermal stability, very low vapor pressure, and high microwave absorption, ionic liquids have attracted great attention as solvents for the synthesis of nanomaterials, being considered as greener alternatives to traditional solvents. While usual solvents often need additives like surfactants, polymers, or other ligands to avoid nanoparticle coalescence, some ionic liquids can stabilize nanoparticles in dispersion without any additive. In order to quantify how the ionic liquids can affect both the aggregation thermodynamics and kinetics, molecular dynamics simulations were performed to simulate the evolution of concentrated dispersions and to compute the potential of mean force between nanoparticles of both hydrophilic and hydrophobic natures in two imidazolium-based ionic liquids, which differ from each other by the length of the cation alkyl group. Depending on the nature of the nanoparticle, structured layers of the polar and apolar regions of the ionic liquid can be formed close to its surface, and those layers lead to activation barriers for dispersed particles to get in contact. If the alkyl group of the ionic liquid is long enough to lead to domain segregation between the ionic and apolar portions of the solvent, the layered structure around the particle becomes more structured and propagates several nanometers away from its surface. This leads to stronger barriers close to the contact and also multiple barriers at larger distances that result from the unfavorable superposition of solvent layers of opposing nature when the nanoparticles approach each other. Those long-range solvent-mediated forces not only provide kinetic stability to dispersions but also affect their dynamics and lead to a long-range ordering between dispersed particles that can be explored as a template for the synthesis of complex materials.

## Introduction

Ionic liquids (ILs) are defined as salts with melting points below 100 °C, with many of them being liquids in a broad temperature range including room conditions. Those low melting points are obtained by the presence of bulk, flexible, and low-symmetry ions, which weaken the ionic interactions and increase the entropy gain upon melting.^1−4^ Usually, the cation derives from an organic molecule, like the imidazolium-, ammonium-, and phosphonium-based cations, and the anion can be both organic, like acetate and ethyl sulfate, or inorganic, like tetrafluoroborate and hexafluorophosphate. This composition makes ILs good solvents for both organic and inorganic species and, due to their essentially null vapor pressure and usually high thermal and chemical stability, they are often claimed to be greener alternatives to the traditional solvents for both synthesis and extraction,^5,6^ although not every IL can be considered green, with some being toxic or presenting limited thermal stability.^7^ The physical and chemical properties of ILs are affected by both the cation and the anion, which can lead to more than 10^12^ combinations. This renders the ILs the title of design solvents since, in principle, their properties can be fine-tuned for a given application by proper choice or modifications in the ions.^8−10^

Recently, there is a growing interest in the use of ILs in the synthesis of nanomaterials, with the imidazolium-based ILs being the ones most used so far.^10−12^ In addition to the low volatility and thermal stability, which enables the use of higher temperatures than most usual solvents, they present high microwave absorbance, which enables ultrafast microwave-assisted synthesis of nanoparticles (NPs).^13−15^ The exchange of the cation or the anion of the IL can also provide additional control in addition to the physical conditions. It was observed, for instance, that the anion affects the size of Ag NPs in imidazolium-based ILs^16^ and that the anion is more likely to affect the stability of Au NPs,^17^ besides changes in the cation structure can also affect the size and shape of the Au and Pt NPs.^18^

Among several interesting properties of ILs in the synthesis of NPs, of particular interest in this work is the capacity that many ILs present to stabilize NPs and other nanomaterials like carbon nanotubes^19^ in dispersions without the use of additives like polymers,^20^ surfactants,^21,22^ or grafted organic molecules over their surface,^23^ which are often needed in other solvents to avoid the contact and sintering between NPs. This stability cannot be attributed to a conventional electric double-layer formation around the particle, since the high concentration of ions in ILs and related solvents would result in a Debye screening length of only ca. 1 Å for the diffuse layer, resulting in a double-layer interaction too weak (if any) to compete with the van der Waals attraction between NPs.^24^ Instead, this stabilization is usually attributed to the formation of alternated layers of ions around the NPs.^25,26^ Those layered structures can be a result of a charge alternation with the formation of a preferred layer of cation or anion close to the particle surface followed by a layer of the opposite ion. Solvent charge alternation was used to explain the stabilization of NP dispersions not only in ILs but also in molten eutectic mixtures of salts composed by smaller ions like NaSCN/KSCN.^24^ This charge alternation manifests usually as a short-range structure resulting from the electroneutrality criteria. A different kind of layered structure can be formed by the segregation between hydrophilic and hydrophobic portions of IL, where regions of low charge density, like alkyl groups, which interact mostly by weak London dispersion forces, are expelled from the contact with high charge-density portions of the cation and the anion, which interacts by strong ionic interactions.^27−29^ In both cases, solvent layers of opposing natures (cation-rich and anion-rich layers in the case of charge alternation or hydrophilic and hydrophobic layers in ILs with an amphiphilic nature) ordered around each NP may be superimposed as the NPs approach each other. The unfavorable mixture of solvent layers of opposing natures can lead to a solvent-mediated repulsion between the NPs and stabilize the dispersion.

The hydrophobic/hydrophilic or polar/apolar domain segregation in ILs is observed if the alkyl chain is long enough^28^ and is characterized experimentally by X-ray or neutron scattering^30−32^ and rheology,^33^ as well as observed in computer simulations.^27,34,35^ The strength of the segregation and the size of the domains tend to increase with the cation alkyl group length and so is the stabilization provided to an NP dispersion. Atomic force microscopy showed the presence of an oscillatory force between mica surfaces separated by films of both 1-butyl-3-methylimidazolium bis(trifluoromethylsulfonyl)imide, [BMIM][NTf_2_], and 1-hexyl-3-methylimidazolium bis(trifluoromethylsulfonyl)imide, [HMIM][NTf_2_], with both the intensity of the force peaks and the distance between peaks increasing as the cation alkyl group length increases.^28^ It was also observed that the increase of the alkyl group of the cation improves the stability of dispersions of both metallic^36^ and ceramic^37,38^ NPs in ILs. Besides the stabilization of nanomaterials, the formation of domains in ILs can also be explored as a template for the synthesis of nanoporous materials, providing smaller and more reproducible pore size than similar methods in surfactant solutions.^11^

Despite the undeniable importance of the hydrophobic/hydrophilic domain segregation in ILs for the stabilization of colloidal dispersions, it is not fully understood yet how this effect affects the interaction between NPs as well as the importance of specific interactions between the particle surface and the ions for the stabilization.^12^ Molecular dynamics simulations, Monte Carlo simulations, and quantum chemical calculations had been employed for some years to elucidate the organization and interactions of ILs close to NPs,^17,24,39−43^ nanotubes,^44,45^ and solid substrates.^29,44,46−49^ However, to the best of our knowledge, no computer simulation work provided a direct calculation of how the domain segregation and the interaction between IL and NP modulates the aggregation thermodynamics and the kinetic stability of NP dispersions since most of them consist of simulations with only one NP or only one solid substrate interacting with the IL. Some exceptions are the works of Frost et al.^41^ and of Khavani et al.^43^ where molecular dynamics simulations were performed with several NPs and provided qualitative information regarding the kinetic stability of the dispersions. Still, no quantitative determination of the free energy or the activation barriers for the aggregation were obtained. Also, the work of Khavani et al. studied the use of ILs as an additive in aqueous dispersions of NPs instead of as the solvent, showing that the coating with amino acid-based ILs prevents the contact between Au NP cores.

In the present work, the potential of mean force was computed between pairs of NPs in two ILs that differ by the length of the cation alkyl chain: 1-butyl-3-methyl-imidazolium tetrafluoroborate ([BMIM][BF_4_] or C4) and 1-octyl-3-methyl-imidazolium tetrafluoroborate ([OMIM][BF_4_] or C8) and concentrated dispersions with several NPs were simulated with microsecond time scale simulations. To access how the interaction between the NPs and the IL affects the colloidal stability, two different models were compared for the particle: one NP that interacts well with the charged portion of the IL (imidazolium ring of the cation and the BF_4_^–^ anion), which will be called the hydrophilic NP, and another NP that interacts better with the alkyl groups of the cation, hence being called the hydrophobic NP. To limit the effects involved, both NP models were generated with the same crystalline structure, size, and mass, with the only difference being the interaction parameters with the IL (see details in the Methods section). Although those models do not intend to reproduce exactly any realistic NP, the hydrophilic one will present a behavior similar to that of a hydroxylated ceramic NP, and the hydrophobic NP will behave as expected by a NP with the surface covered by CH_3_ groups. Only electrically neutral NPs will be discussed here, with the effect of a net charge on the NPs being more suitable for future work since it is expected to add additional complexity to the problem. In the next section, the results will be presented and discussed, starting with a discussion regarding the thermodynamics for the association between 2 NPs in both ILs and how the solvent organization around the NPs can induce activation barriers for aggregation. After that, the behavior of the NPs in dispersions with high concentrations in the same ILs will be discussed, showing how the results obtained for the association between a pair of NPs can be used to understand the collective behavior in systems with many NPs inducing organization in the solvent and interacting with each other.

## Results and Discussion

### Solvent Organization and Association Thermodynamics

**Figure 1:**
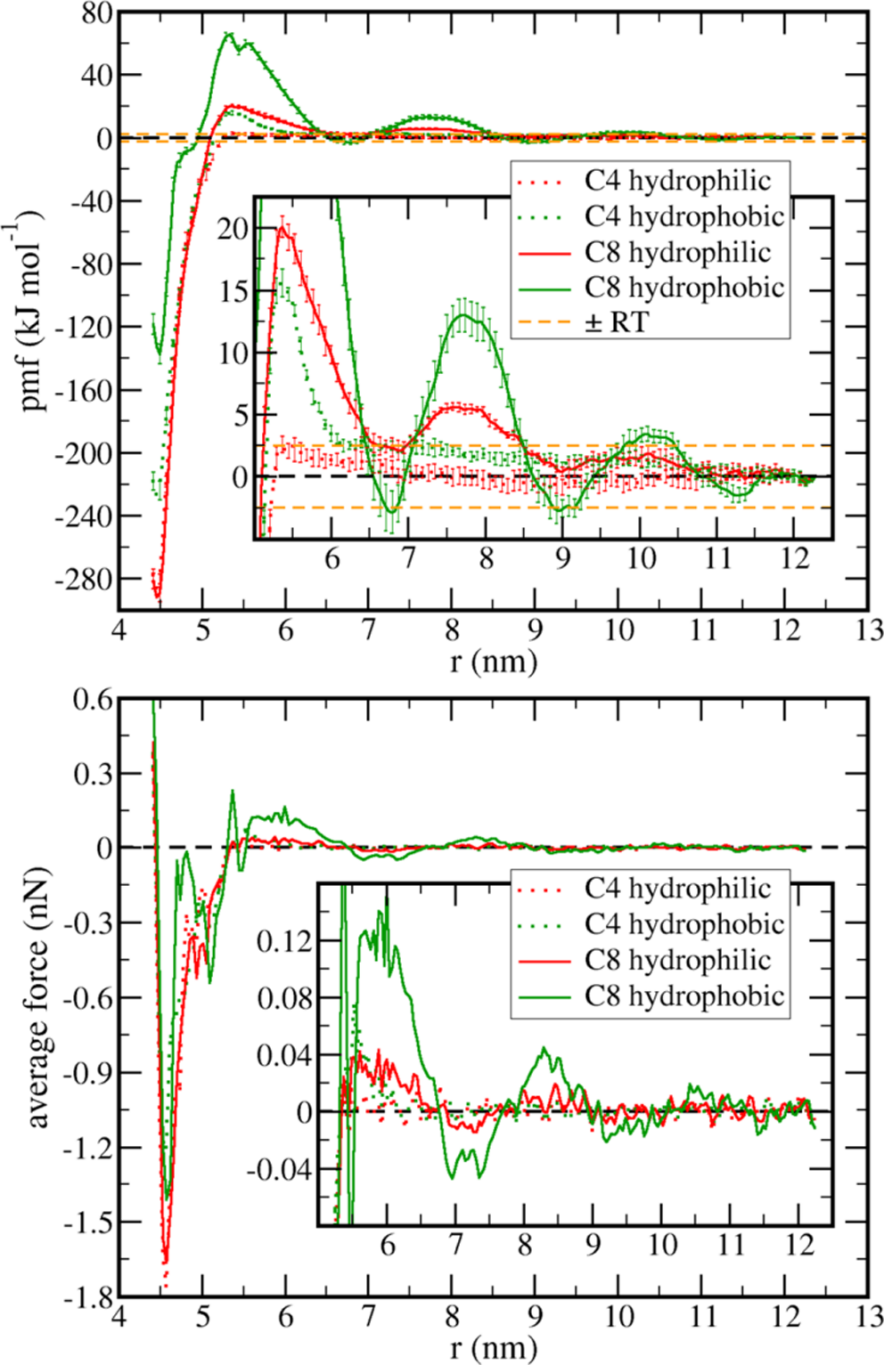
Top: potential of mean force as a function of the distance *r* between the centers of mass of two nanoparticles for the dissociation processes. Bottom: average force acting along the reaction coordinate. Different colors stand for different nanoparticle natures: hydrophilic (red curves) and hydrophobic (green curves). Different line styles indicate the ionic liquid used as the solvent: 1-butyl-3-methylimidazolium tetrafluoroborate (C4, dotted curves) and 1-octyl-3-methylimidazolium tetrafluoroborate (C8, solid curves). The insets zoom in on the long-range behavior.

In order to obtain the aggregation free energy (Δ_ag_*G*) for a pair of NPs in ILs and also to characterize free-energy barriers for the aggregation, the potential of mean force (*pmf*) was computed (Figure 1, top) for the dissociation of both hydrophilic and hydrophobic particles (red and green curves, respectively) in both 1-butyl-3-methyl-imidazolium tetrafluoroborate ([BMIM][BF_4_] or C4, dotted curves) and 1-octyl-3-methyl-imidazolium tetrafluoroborate ([OMIM][BF_4_] or C8, solid curves). The *pmf* corresponds to the reversible work involved when two NPs approach each other; hence, the difference between the value of the *pmf* at two given distances *r*_1_ and *r*_2_ corresponds to the free-energy variation in changing the distance from *r*_1_ to *r*_2_. Therefore, local minima correspond to thermodynamically favorable distances, and the maxima correspond to activation barriers. From the *pmf* gradient eq 1, we can also derive the average force acting along the reaction coordinate at each distance *r* between NP centers of mass (Figure 1, bottom), which would be the result obtained in an atomic force microscopy experiment if the two NPs were pulled apart from each other inside the solution with a rate slow enough to guarantee that the process is nearly reversible. The force profiles obtained for C8 are qualitatively similar to the atomic force microscopy profiles reported between mica surfaces separated by IL films,^28^ but a quantitative comparison is not possible because the ILs used are not the same and here we are studying small spherical NPs instead of surfaces. Notice that the force profile is noisier than the *pmf* because the numerical calculation of derivatives increases the effect of random noise; thus, we will focus the discussion mostly on the free-energy profile rather than the average force
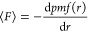
1

The dimer formation is thermodynamically favorable for both hydrophobic and hydrophilic particles in both C4 and C8 ILs with the *pmf* global minimum at 4.46 nm, which corresponds to the sum of the NP radius. The Δ_ag_*G* is more negative for the hydrophilic NPs than for the hydrophobic NPs studied here, and while there is an increase of 90 kJ/mol in the Δ_ag_*G* of hydrophobic NPs by increasing the size of the aliphatic chain of the IL cation, the Δ_ag_*G* of the hydrophilic NPs is almost unaffected by the solvent change. It is important to notice that the stronger thermodynamic tendency of aggregation of the hydrophilic NPs in those ILs does not imply a weak interaction with the solvent. In fact, the hydrophilic NP presents a more negative interaction energy with the ILs (Table S1), especially due to the strong interaction with the anion. However, the NP–NP interaction energy is stronger for hydrophilic NPs than for hydrophobic ones and the hydrophilic particle interacts preferentially with the anion and the polar portion of the cation, which also interacts very strongly with each other by the Coulomb interactions. On the other hand, the hydrophobic NPs interact preferentially with the alkyl groups of the IL cation, which displays weaker dispersion interactions inside the liquid. Hence, the solvation of the hydrophobic particles results in a smaller interaction loss in the IL contributing to a less negative Δ_ag_*G*.

**Figure 2:**
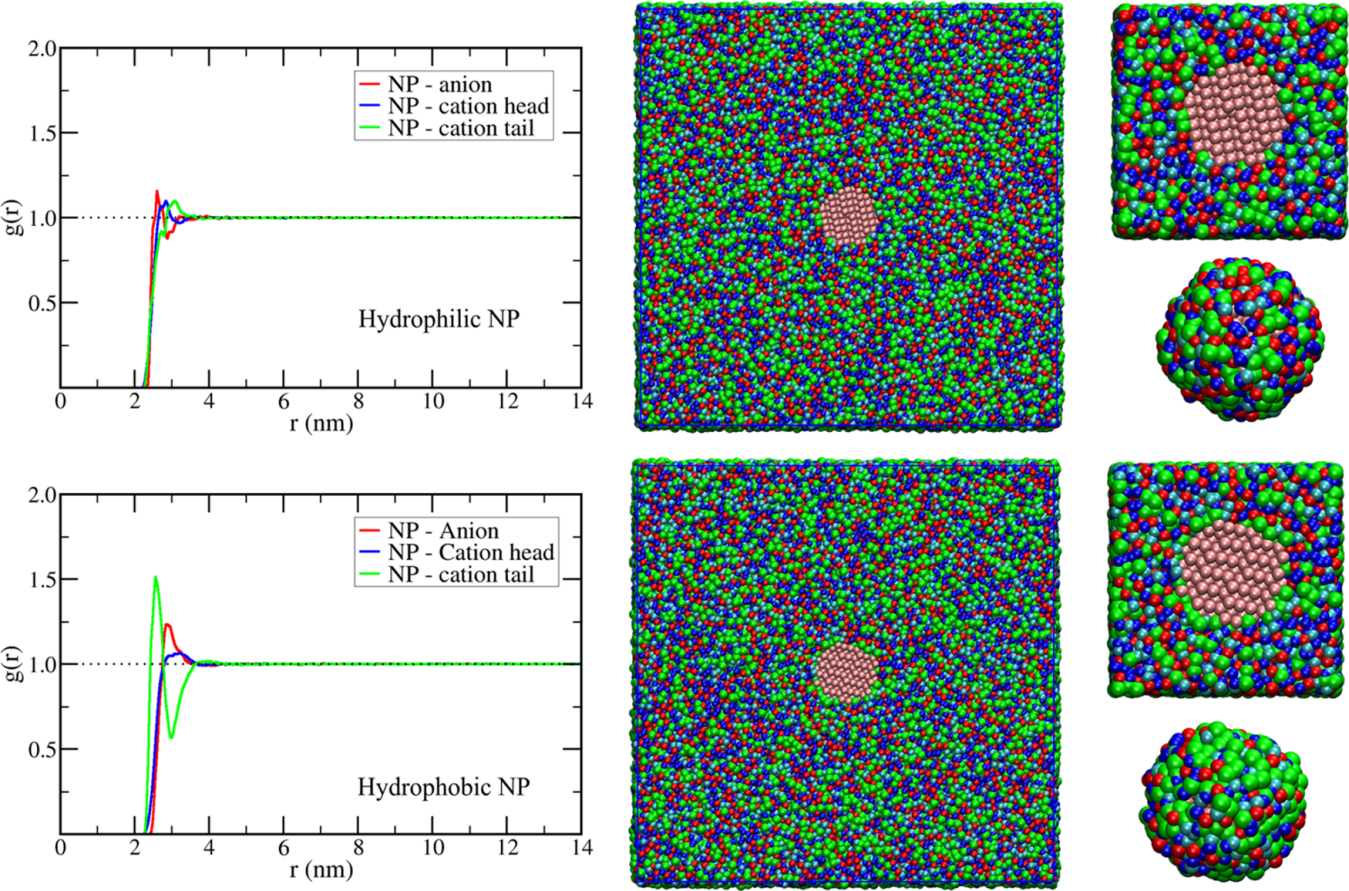
Structure of the solvent around hydrophilic (top) and hydrophobic (bottom) NPs in the ionic liquid 1-butyl-3-methyl-imidazolium tetrafluoroborate (C4) Left: radial distribution function of anion, cation imidazolium group, and cation alkyl group around the NP center of mass. Middle: graphical representations of the final structure showing a transversal slice of the whole simulation box with the NP in the center. Right: zoom of the transversal slice close to the NP and a representation of the first shell (0.6 nm from any NP site) of interaction sites around the NP, with sites of the polar portion of the cation shown in blue (cyan for the uncharged site and dark blue for the charged ones), green for the hydrophobic portion of the cation, and red for the anions.

Besides the aggregation being thermodynamically favorable in both C4 and C8 ionic liquids, there are energy barriers for the dimer association larger than the thermal energy, except for the hydrophilic NPs in C4 (Figure 1, top). The increase of the cation alkyl group from butyl to octyl not only dramatically increases the barrier prior to the contact minimum but also results in a complex pattern for the long-range behavior of the *pmf*, with multiple maxima and minima. It is important to notice that the direct interaction between NPs is given by a Lennard-Jones potential (eq S1 in the Supporting Information; parameters in Table S2); hence, this is a short-range interaction and becomes negligible for *r* > 6 nm, implying that those multiple barriers are due to solvent-mediated interactions. To characterize those, the structure of the solvent around NPs was analyzed using the radial distribution function of the anion, the cation polar head, and the cation hydrophobic tail in model systems containing only one NP in the solvent. The radial distribution function is given by the density of a species of interest at a spherical shell with radius *r*_AB_ around the NP center of mass divided by the average density in the whole system eq 2. Figure 2 shows the results for the solvent organization around the NPs in C4. Around the hydrophobic particle, as expected, there is an excess of cation apolar tail (green curve in *g*(*r*) and green spheres in the structure representations at Figure 2, bottom), and this is followed by a region rich in the polar portion of the ionic liquid (red and blue curves and spheres). The opposite is found for the hydrophilic particle, which prefers to interact with the polar portions of the ionic liquid. However, as noticed by the radial distribution function curves, the solvent organization around the hydrophilic NP is weaker than around the hydrophobic one. As discussed above, this does not imply in a stronger NP–solvent interaction for the hydrophobic one; instead, the weaker organization around the hydrophilic NP results from the strength of the ionic interactions between the cation head and the anion, which made those groups less available to solvate the hydrophilic particle
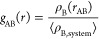
2The weaker solvent structure close to the NP is not a general feature of hydrophilic NPs; instead, it is a consequence of the interaction parameters employed. Increasing the interaction between hydrophilic NP sites and the anion by 50% leads to the distributions and structures shown in Figure S1 in the Supporting Information file, which displays peaks with similar intensity displayed by the hydrophobic NP but with the first layer rich in anions instead. This change in the interaction parameters, although arbitrary in the simulation, could correspond to a change in either the NP material or the IL anion. The results of Figure S1 are more likely to reproduce a metallic NP than the parameters employed for the hydrophilic NP for the rest of this work since metallic NPs tend to interact preferentially with the anion of ILs.^17,40^ If *pmf* was computed for the NP of Figure S1, a higher activation barrier would be observed for the hydrophilic NPs in C4. The “weaker” or “stronger” structure of a given IL close to an NP is not necessarily a consequence of the hydrophilic or hydrophobic nature but instead of the relative intensity of the interaction of the NP with specific groups of the IL in relation to their interactions inside the IL.

**Figure 3:**
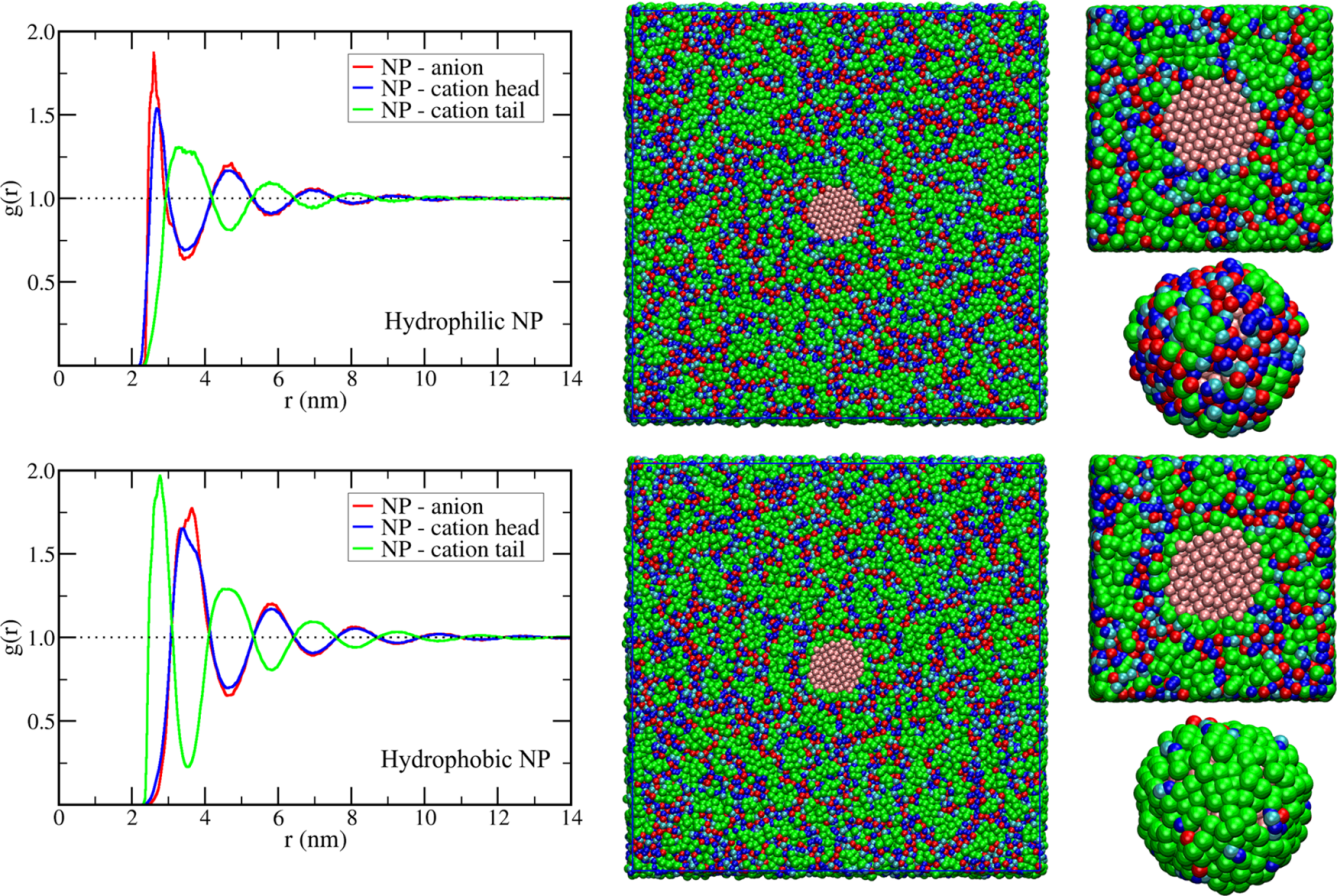
Structure of the solvent around hydrophilic (top) and hydrophobic (bottom) NPs in the ionic liquid 1-octyl-3-methyl-imidazolium tetrafluoroborate (C8) Left: radial distribution function of anion, cation imidazolium group, and cation alkyl group around NP center of mass. Middle: graphical representations of the final structure showing a transversal slice of the whole simulation box with the NP in the center. Right: zoom of the transversal slice close to the NP and a representation of the first shell (0.6 nm from any NP site) of interaction sites around the NP, with sites of the polar portion of the cation shown in blue (cyan for the uncharged site and dark blue for the charged ones), green for the hydrophobic portion of the cation, and red for the anions.

The C4 solvent around both NPs displays only a short-range ordering, as can be seen by the *g*(*r*) achieving an ideal value of 1 just after two solvation layers (considering here one layer due to the hydrophobic and another due to the hydrophilic portion of the IL). The solvent structure becomes more interesting in the C8 solvent (Figure 3). First, the longer alkyl group makes the first layer more structured even for the hydrophilic NP, which results in the increase of hydrophilic NP–anion interaction energy in C8 in comparison to C4 (Table S1). Also, the longer alkyl group leads to domain segregation^27,28,34^ between the polar and apolar portions of the ionic liquid (Figure 3).

**Figure 4:**
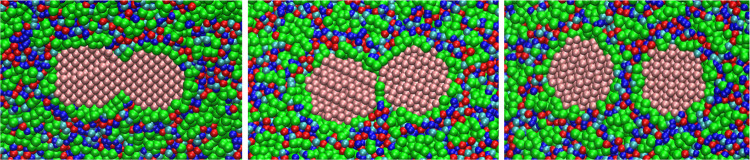
Snapshots of some structures obtained along the calculation of *pmf* between a pair of hydrophobic NPs in the C8 solvent. From left to right: at the *pmf* global minimum (*r* = 4.6 nm), at the first barrier top (*r* = 5.3 nm), and at the second minimum (*r* = 6.7 nm). Color scheme is the same as that used in Figures 2 and 3

The presence of those domains leads to a long-range organization of the solvent with alternating hydrophilic and hydrophobic shells around NPs that persist up to 12 nm from the NP center, as can be seen by the radial distribution functions in Figure 3. Those alternated layers around NPs result in the complex long-range behavior of *pmfs* in the C8 IL: as the two NPs approach, there is a superposition of the solvent layers around them. Depending on the distance between the particles, there may be a superposition between hydrophobic and hydrophilic layers, resulting in a solvent-mediated repulsion that results in the local maxima of the *pmfs*, or there may be a superposition of the same type layers resulting in the local minima of the *pmfs* (solid curves in Figure 1). This can be seen by the graphical representation of selected structures along the *pmf* calculations in C8 (Figure 4), where we can notice some mixture of polar and apolar sites of the IL between NPs in the position of the first maximum and the formation of more well-defined polar and apolar layers between them when the distance corresponds to the minima of the *pmf*. The distance between layers of opposing natures in the *g*(*r*) curves of Figure 3 stands for ca. 2.1 nm; thus, if at a given distance between NPs there is a superposition of layers of the same kind, moving one particle either closer or farther to the other leads to an unfavorable superposition of hydrophilic and hydrophobic layers, which results in the separation of consecutive maxima and minima points of the *pmf* (Figure 1) also being of ca. 2.1 nm. Those distances are the same for both hydrophilic and hydrophobic particles, indicating that the relative positions of the maxima and minima in the *pmf* are governed by the size of the domains formed in the solvent and do not depend on the nature or strength of the interaction between the NP and the solvent. However, the NP–solvent interaction affects the intensity of the solvent structure around NPs, which, in turn, defines the height of the barriers in the *pmf*. In the models studied here, the hydrophobic NP induces a stronger organization of the C8 IL than the hydrophilic particle and, as a consequence, presents higher barriers for dimer association in the *pmf*.

### NP Aggregation in Concentrated Dispersions

**Figure 5:**
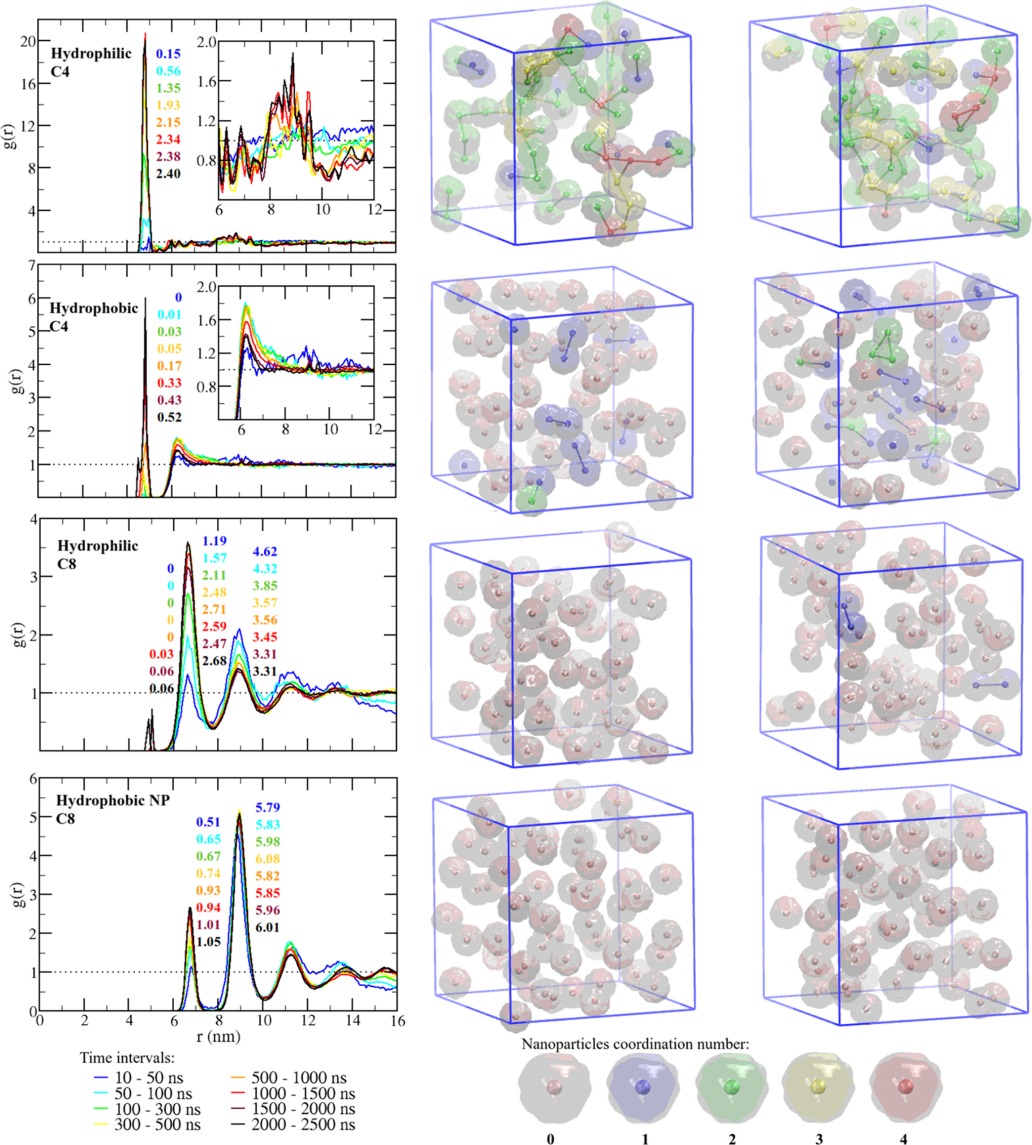
NP organization in high-concentration dispersions in C4 (top) and C8 (bottom) ionic liquids. Left: radial distribution function between NP centers of mass at different time intervals given by different colors and the coordination number corresponding to each peak indicated by the figures close to it. Right: snapshots from the simulations at 1000 and 2500 ns showing each NP colored according to the number of NPs in contact with it. The center of mass of each NP is shown as a solid sphere, while the surface area of the NP is represented as a translucid surface. Bonds were placed between the centers of mass of two NPs if the distance between them is equal to or smaller than 5.1 nm, the position of *g*(*r*) first minimum.

In the previous section, we discussed how the NP–IL interaction and the domain segregation in the solvent control the association of two NPs forming a dimer. This section will focus on how the same variables affect the collective behavior in a dispersion containing several NPs in a high NP concentration of ca. 0.0026 mol/L. Starting from a distance of 8.4 nm between the center of mass of the closest NPs, the evolution of the system was monitored by 2500 ns by computing the radial distribution function between the center of mass of NPs at several time intervals (left of Figure 5, where curves of different colors correspond to different time intervals) and by the interaction energy between NPs (Figures S2 and S3 in the Supporting Information file). As stated in the previous section, the aggregation is thermodynamically favorable, and for the dispersions in C4 (top 2 panels in Figure 5), it is noticeable the appearance of a peak in the contact distance (between 4.5 and 5.0 nm) in less than 100 ns and its fast increase as more NPs get into contact.

The average number of NPs in the distance corresponding to each peak can be computed by the integration of the *g*(*r*) peak (eq 3, where *r*_1_ and *r*_2_ are the positions of the minima before and after the maximum of interest), and those values were included close to the corresponding peaks of *g*(*r*) in Figure 5 for each time interval. Comparing the hydrophobic and the hydrophilic NPs in C4, one can notice that the aggregation is faster for the hydrophilic NP, which displays the smallest barrier for the dimer association across the four systems studied (Figure 1). After 1000 ns, every hydrophilic NP in the dispersion in C4 is in contact with at least one other NP, and most of them present at least 2 neighbors after 2500 ns (structures in the middle and right of Figure 5, where NPs were colored differently according to the number of other NPs in contact to them). In this system, even before 800 ns, almost all of the particles are bonded into a single cluster (be aware of the periodic boundary conditions when looking at those structures: NPs close to one edge of the simulation box may be in contact with another particle in the opposite side of the box).
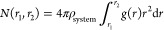
3

In the dispersion of hydrophobic NPs in the same solvent (second row of Figure 5), due to the high barrier for the association, even after 2500 ns, most of the NPs are still dissociated. Instead of a large cluster, only some dimers and trimers of NPs were observed and the average number of neighbors stayed below 1. The same conclusions can be achieved by the analyses of the NP–NP interaction energy evolution in both dispersions (Figures S2 and S3), which display a faster decrease for hydrophilic than for hydrophobic NPs. However, care must be taken when comparing the variation of the NP–NP interaction: while the most important effect is the faster aggregation, the interaction energy for each contact is more negative for hydrophilic particles than for the hydrophobic particles (Table S1). Is important to notice that this simulation does not achieve an equilibrium state, with the first *g*(*r*) peak and the corresponding coordination number still increasing; however, those results already show a stronger kinetic stabilization due to the stronger solvent organization around the hydrophobic NPs in comparison to the hydrophilic NPs. As noticed in the previous section, the differences pointed between the hydrophobic and the hydrophilic models are a consequence of NPs being able to produce organized layers of the ILs close to its surface and, depending on the NP–IL interactions, a dispersion of hydrophilic NPs can be more stable than the one of hydrophobic NPs.

Before moving forward to the dispersions in the C8 solvent, another interesting distinction can be noticed between the two dispersions in C4 in the long-range behavior of the *g*(*r*) curves. Both dispersions present a secondary *g*(*r*) maximum but with different shapes, intensities, and positions. For the hydrophobic particle, the position slightly after 6 nm is consistent with 2 NPs separated by a solvent layer and matches the end of the free-energy barrier noticed on the respective *pmf* (Figure 1). A similar peak due to NPs separated by a solvent layer is also noticed for both NPs in C8; however, for the hydrophilic particle in C4, the solvent layers are ill-defined, and there is no significant barrier for the dimer association, which explains the absence of the maximum separated by a solvent layer around 6.0 nm. On the other hand, there is a broad maximum around 9.0 nm, which is nearly 4 times the radius of the NP and corresponds to the distance between second neighbors in the cluster formed if the angle between three bonded NPs is close to 180°. The broadness of this maximum, however, indicates that the cluster formed is not much structured beyond the first neighbors around each particle.

**Figure 6:**
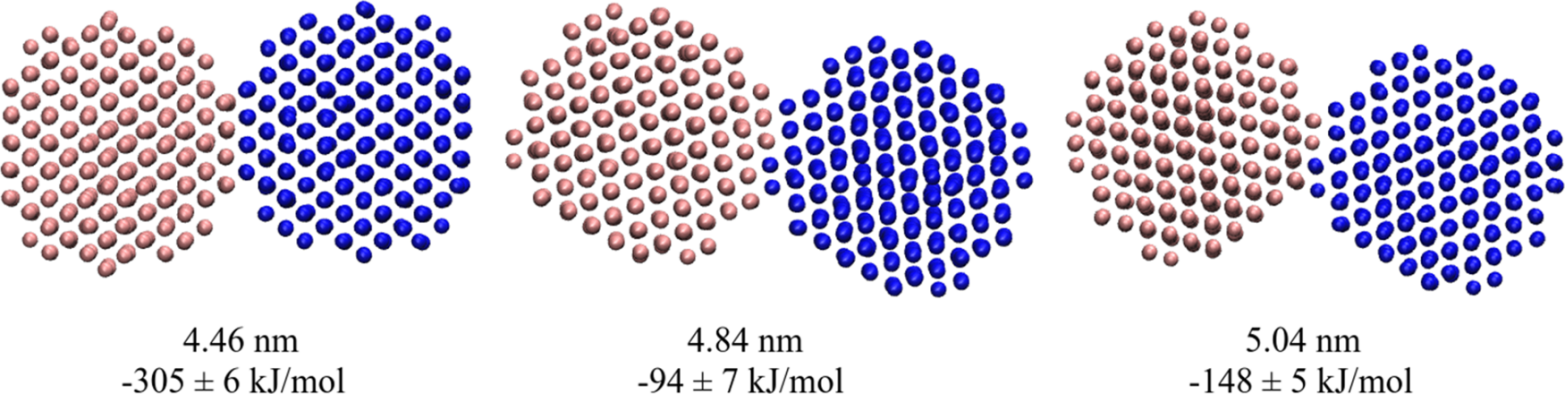
Dimer that corresponds to the minimum of the potential of mean force (left) and dimers formed in the simulation of hydrophilic NP dispersion in C8 (middle and right), with the distance between the center of mass of the two NPs and the computed interaction energy between them given below the respective structures.

The contact peak happens at a distance slightly larger than the position of the *pmf* global minimum, and as noticed in the distribution of hydrophobic NPs in C4 and of hydrophilic NPs in C8, it is composed of two very close peaks that are almost superimposed. Those features result from contacts between different faces of the NPs as shown in Figure 6. The *pmf* calculations started from the dimer with the maximum number of contacts (left of Figure 6), which renders the stronger interaction between the NPs and a distance between centers of mass of 4.46 nm, the position of the *pmf* global minimum. However, this structure was not formed spontaneously in the simulations of the NP dispersions; instead, dimers with slightly larger distances between particle centers were formed (middle and right of Figure 6), rendering the *g*(*r*) first peak shift to larger distances in comparison to the *pmf* minima.

With the increase in the alkyl chain of the IL cation (two bottom rows of Figure 5), the radial distribution function curves start to display several peaks that match the positions of the corresponding *pmf* minima. For the hydrophilic NP dispersion in C8, no aggregation was observed up to 1000 ns of simulation, a time at which every NP was bonded to at least one other in the corresponding case in C4. Even at 2500, only 2 dimers were formed from a system with 64 NPs. This results in only very small variations of the NP–NP interaction energy across the simulation when compared to the dispersion of the same NPs in C4 (Figure S2). Although this simulation is not long enough to reach an equilibrium state, at which certainly every particle would be aggregated since the Δ_ag_*G* is very negative, the *g*(*r*) patterns change very slowly after 1000 ns, indicating that no huge change would be expected until simulation times orders of magnitude larger than performed here. Notice that simulations with coarse-grained force fields as the ones performed here tend to result in faster dynamics than in real world due to the smooth potential surface in the coarse-grained models, which is also noticed by the underestimated zero shear viscosity of the C4 obtained with the same force field.^50,51^ Hence, in the real world, the kinetic stabilization of similar NP dispersions is likely to be even greater.

Finally, for the dispersion of hydrophobic NPs in C8, no aggregation occurs even after 2500 ns, which is due to the larger barriers resulting from the stronger solvent domain organization around NPs in this system. Not only were no dimers formed, but no nanoparticles even entered the cutoff radius of another for calculating interactions. Hence, the contribution of the NP–NP interaction for the system energy was zero along the whole simulation (Figure S3). As a consequence, no peak at the contact distance is noticed in the *g*(*r*) curves for this system, and even the approximation up to a distance of ca. 6.5 nm where only one solvation layer separates the NPs is a slow process, as can be seen by the peak at 6.0 nm being smaller than those at ca. 9.0 nm along the whole simulation of this system. In comparison, for the hydrophilic NPs in the same solvent, this trend was inverted already at 100 ns. This shows that not only the 65 kJ/mol major barrier in this system provides a strong kinetic stabilization but also the secondary barrier slows down the approximation between NPs. In fact, the secondary barrier for the hydrophobic NPs in the C8 solvent is as high as the only barrier for the aggregation of the same NPs in C4 (Figure 1).

**Figure 7:**
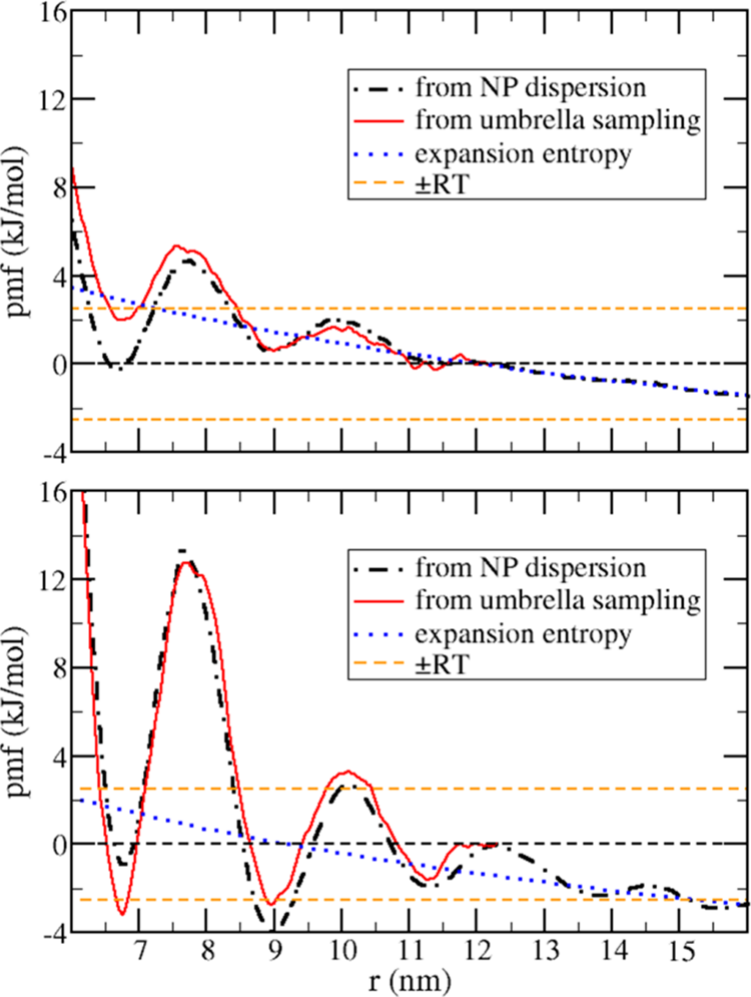
Comparison of the potential of mean force computed from the radial pair distribution function between NPs in concentrated dispersion and the long-range behavior of the potential of mean force computed with umbrella sampling simulation between a pair of NPs in a C8 ionic liquid. Top: hydrophilic NP, bottom: hydrophobic NP.

Despite the positions of maxima and minima of the NP–NP radial distribution functions for the dispersions in C8 agreeing well with the positions of the extreme points in the *pmf*, one can question how much the *pmf* computed for a dimer of NPs can predict the distribution in a concentrated dispersion and vice versa. To tackle the question, the *pmf* was computed from the radial distribution functions between NPs in the dispersions in the C8 IL at the last time interval (2000–2500 ns, black curves in Figure 5) using eq 4, where *T* is the temperature and *R* is the ideal gas constant. The second term in eq 4 stands for the entropy variation associated with the change in the available volume when moving from a spherical shell of radius *r*_0_ to a spherical shell of radius *r*eq 5,^52^ a contribution that needs to be included in the *pmf* computed from the radial distribution function. Due to the poor sampling at the contact region in the dispersion of the hydrophilic particle and its total absence in the case of hydrophobic particles, only the region after the first maximum of the *pmf* was considered and the results are shown in Figure 7, with all of the curves shifted to give a value of zero at 12.30, the same reference used for the *pmfs* in Figure 1. The *pmf* computed from the NP–NP distribution in the dispersion (black curve in Figure 7) matches very well the long-range behavior of the dimer *pmf*, showing that the long-range solvent-mediated interaction characterized for the dimer remains essentially the same in a dispersion containing several NPs. The agreement is worse for smaller values of *r* due to the fact that dispersions do not reach equilibrium even after 2500 ns and the *g*(*r*) peaks at those positions are slowly changing intensity (Figure 5). The effect of solvent domain segregation tends to become less relevant at large distances, and eventually, the behavior is dominated by the volumetric entropic term eq 5
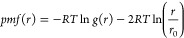
4
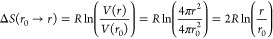
5

**Figure 8:**
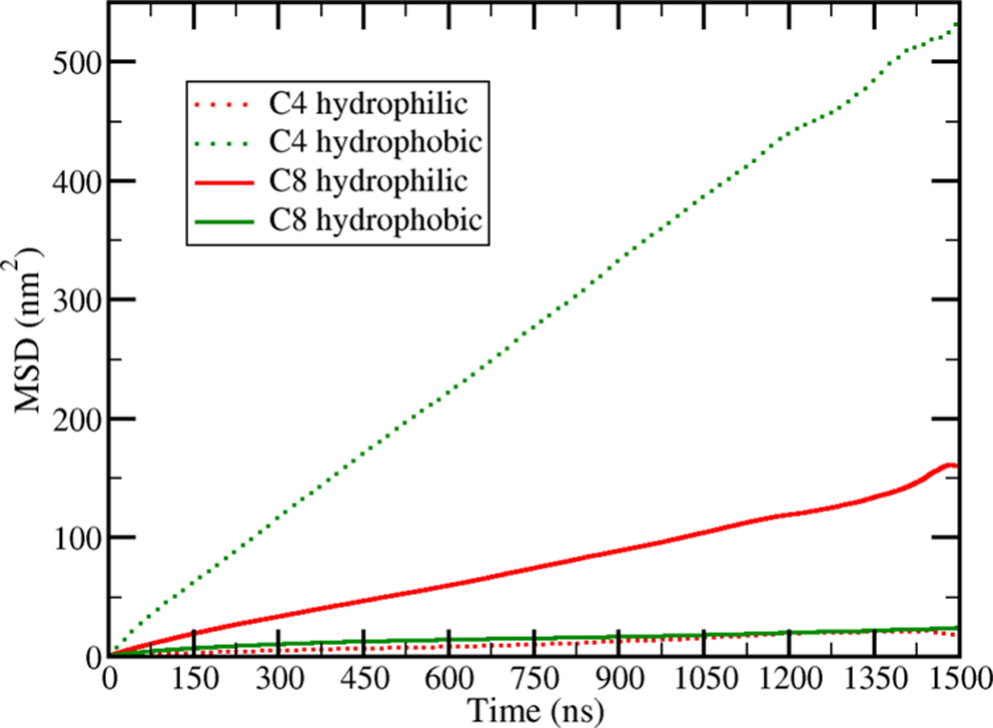
Mean square displacement (MSD) of nanoparticles in the concentrated dispersions computed after 1000 ns.

The long-range forces and the aggregation between NPs also affect their dynamics in the dispersion, which can be analyzed by the mean square displacement (MSD; Figure 8). For the hydrophilic NP (red curves), the MSD increases faster in C8 than in C4, indicating faster dynamics in the former. However, the opposite trend is noticed for the hydrophobic particles (green curves). The major effect for the hydrophilic particles is the aggregation into a single large cluster in the C4 solvent (Figure 5), which strongly restricts the NP motion, while in C8, only a few dimers were formed. On the other hand, for the hydrophobic particles, only dimers and trimers were formed in C4, enabling fast dynamics in this solvent, while the solvent-mediated forces in C8 are strong even at large distances, resulting in slow dynamics. All in all, the system with almost no preferential solvent structure that leads to a large cluster and the one with strong solvent structuration, which avoids any clustering, present both very similar MSD and slow NP dynamics.

## Conclusions

The segregation between polar and apolar domains generates multiple activation barriers for the aggregation of nanoparticles in ionic liquids due to the unfavorable superposition of layers of opposite nature when the particles get closer to each other. The height of the main barrier before contact can be larger than 60 kJ/mol, and even secondary barriers can be higher than the thermal energy. Those barriers provide kinetic stability to colloidal dispersions in ionic liquids without the need for additives.

The distance between the consecutive barriers depends only on the size of the domains formed in the liquid, which can be controlled by the size of the alkyl group of the cation. On the other hand, the height of those activation barriers depends on the nature of the NPs and their interactions with the ionic liquid, with particles that induce stronger organization of the ions at their surface presenting also more structured layers even at some nanometers away from the surface and, consequently, higher activation barriers and stronger stabilization of the dispersions. However, a stronger interaction with one specific portion of the ionic liquid may not be enough to guarantee a stronger organization of the solvent since the interaction between the solvent ions also needs to be taken into account, especially the interaction between the oppositely charged groups, which are strong and compete with the solvation of the particle surface, which can lead to a weak structure of the liquid close to the particle surface and smaller stabilization.

The multiple barriers not only prevent the contact between NPs and provide kinetic stability to the dispersion, but also affect their dynamics and induce a long-range organization of nanoparticles. The slowdown of nanoparticle dynamics can potentially be explored to produce gels, and the long-range ordering can be used as a template for the synthesis of complex materials, with nanocrystals of one material dispersed inside a matrix of a different material, with the spacing between nanocrystals of the dispersed material controlled by the size of the domain segregation in the solvent.

## Methods

### Force Field Parameters and NP Structure

Due to the size of the model system and the simulation time needed to properly characterize the self-assembly and to compute the potentials of mean force (*pmf*), the Martini 3.0 coarse-grained force field^53,54^ was employed in all simulations presented here. In a coarse-grained force field, several atoms are clustered together in a single interaction site, which reduces the number of particles to compute and also enables a reduction in the integration time step of the molecular dynamics (MD) simulation. Two ionic liquids were employed and compared in this work: 1-octyl-3-methylimidazolium tetrafluoroborate ([OMIM][BF_4_] or C8) and 1-butyl-3-methylimidazolium tetrafluoroborate ([BMIM][BF_4_] or C4). In the Martini 3.0, the anion [BF_4_]^−^ is described by a single interaction site and the cations [BMIM]^+^ and [OMIM]^+^ are represented by 4 and 5 sites, respectively, with 3 interaction sites used to describe the charged imidazolium ring and the other sites to describe the aliphatic tail (coarse-grained model represented together with the structural formula in Figure S4). This model was already used to study the viscosity and non-Newtonian behavior of [BMIM][BF_4_] both in bulk and confined between solid surfaces.^50,51^

The nanoparticles were produced by cutting a sphere of 2.2 nm from a face-centered-cubic (FCC) crystal with neighbor atoms distant by 0.53 nm (which corresponds to the minimum of the Lennard-Jones potential between particles of the regular size in the Martini force field) from each other, rendering nearly spherical NPs with 580 interaction sites (left of Figure S5). Harmonic potentials with a force constant of 2500 kJ mol^–1^ nm^–2^ were applied between neighboring sites of the NP to hold its structure. Using the same structure, two different NPs were studied by changing the interaction potential used to describe them, the sites of the hydrophilic NP being described by the type P4 and the ones from the hydrophobic by the type C1. One P4 site presents a transfer free energy between oil and water of ca. −17 kJ/mol, while the C1 presents a value of ca. 17 kJ/mol.^53^ Thus, an NP described by P4 sites would be strongly hydrophilic and can represent a ceramic NP with the surface covered by OH groups, while the NP formed by C1 sites is strongly hydrophobic such as a particle with a surface covered by CH_3_ groups. Interaction parameters between the NPs and the ILs are given in Table S2 in the Supporting Information file. The mass of one gold atom (197 g/mol) was arbitrarily attributed to the NP sites. Below, the preparation of the initial structure for each type of simulation performed is described, with the respective compositions given in Table 1.

**Table 1:** Number of Nanoparticles and Ion Pairs, Final Size of the Simulation Box, and Total Simulation Time in Each Study Performed in This Work As Well As in Which Figures of the Manuscript Each Simulation Was Used

simulation	IL	NPs	ion pairs	final box edge (nm)^a^	simulation time (ns)^b^	used in figures
single NP simulations	C4	1	121,600	33.1	200	2, S1
	C8	1	86,400	33.2	200	3
pmf calculations	C4	2	64,000	26.8	10,284	1, S6, S8
	C8	2	60,000	29.5	10,284	1, 4, 7, S7, S9
concentrated dispersions	C4	64	121,600	34.3	2500	5, 6, 8, S2, S3
	C8	64	86,400	34.3	2500	5, 6, 7, 8, S2, S3

a Up to the first decimal digit, there is no difference in the value of the box edge of the final structure produced in the simulations due to the hydrophilic or hydrophobic nature of the NPs.

b For the pmf calculations, the value given is the sum over every simulation performed to compute one pmf.

### Preparation of Initial Structures for the Simulations

#### Concentrated Dispersions

Initially, a cubic box with an edge length of 8.6 nm, the double of the NP diameter, containing a single NP 1900 C4 or 1350 C8 ion pairs was prepared using the Packmol software.^55^ Those boxes were replicated 4 times in each Cartesian direction rendering cubic boxes with a 34.4 nm edge (structure on the left of Figure S5). The number of ion pairs was selected to result in the same concentration of NPs in both liquids, namely, 0.0026 mol/L after relaxation.

#### Single NP Simulations

Used to characterize the solvent organization around the particle in a dilute regime, those simulations were prepared directly by packing the molecules using the Packmol software with the same amount of solvent used in the simulations of dispersions.

#### Potential of Mean Force Calculations

For the initial structure used in the *pmf* calculations, the Themis software^56^ was used to locate the structure with minimum energy for a pair of NPs in contact (Figure 6) by performing rigid-body rotations and translations of the two NPs and computing the interaction energy using the same parameters employed in the simulations. The dimer with the lowest energy was solvated with ILs using Packmol.

### Simulation Conditions and Software

All of the simulations were performed using the Gromacs 2018.8 software^57,58^ in NPT ensemble with *T* = 300 K maintained through the V-rescale algorithm^59^ with τ_T_ = 1.0 ps and *P* = 1 bar using the Berendsen isotropic barostat^60^ with τ_P_ = 0.5 ps. A cutoff radius of 1.1 nm was used for both Coulomb and Lennard-Jones potentials, with Particle-Mesh Ewald (PME)^61^ correction for long-range electrostatics and a shift potential applied between 0.9 and 1.1 nm to make the Lennard-Jones potential converges smoothly to zero at the cutoff. Also, a relative dielectric constant ε_r_ = 15 was used to attenuate the Coulomb potential, which is done in the Martini force field to compensate the absence of molecular dipoles, which would attenuate the interactions between ions.^53^ An integration time step of 0.02 ps was used in all of the simulations.

The VMD 1.9.3 was used to visualize the trajectories and to render the graphical representations included in this manuscript.^62^

### Potential of Mean Force Calculations

The umbrella sampling method was used to compute the potential of the mean force for the aggregation between pairs of NPs in both ILs. On this method, a bias is introduced to force the sampling of the desired reaction coordinate, in this case, the distance *r* between the centers of mass of the two NPs. To sample all of the distances between the contact distance and the maximum distance enabled by the size of the simulation box, a total of 215 simulations were performed for each *pmf* by changing the minimum *r*_min_ of the harmonic potential used as the bias potential eq 6 from 4.40 to 12.35 nm. Hence, those calculations reproduced the dissociation between the NPs starting from the structure with the lowest energy found for the dimer. Each simulation with the slightest high *r*_min_ started from the final structure of the previous simulation, so the change in the potential corresponds only to a very small perturbation on the system and was performed for 44 ns for *r*_min_ < 6.0 and for 50 ns for *r*_min_ ≥ 6.0, with the first 4 ns of each simulation discarded from analyses. The probability distributions obtained in each simulation were combined by the weighted histogram analysis method (WHAM)^63^ as implemented in Gromacs to generate the potential of mean force
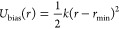
6To guarantee the quality of pmf, all distances along the reaction coordinate must be properly sampled. There must be a good superposition between the histograms of neighbor sampling window in the umbrella sampling method, which was obtained (Figures S6 and S7 in the Supporting Information file). The value of the force constant *k* was adjusted to get a good sampling and different values were needed to be used in different regions of the reaction coordinate. When the two NPs are far from each other (*r*_min_ ≥ 6.0 nm), a weaker force constant *k* = 600 kJ mol^–1^ nm^–2^ could be used and guarantees a proper sampling with a spacing between neighbor windows of 0.05 nm. When they are closer (*r*_min_ ≤ 6.0 nm), the average force is stronger (Figure 1), so a stronger force constant of 3600 kJ mol^–1^ nm^–2^ was used and the separation between the *r*_min_ at neighbor windows was reduced to 0.02 nm. This change was particularly necessary to sample the region of intense attractive forces between the NPs at distances smaller than the first maximum. Even with this stronger bias potential, a few positions of the reaction coordinate displayed a bad sampling and additional simulations were performed with *r*_min_ = 4.605, 4.625, 4.655, 4.675, and 4.725 nm with *k* = 10,000 kJ mol^–1^ nm^–2^. Although this kind of issue is rare in pmf calculations involving only soft matter, like micelles^64^ and lipid bilayers,^65^ which can be performed using a single value of *k* across the whole reaction coordinate, when involving crystalline materials like those NPs, the sampling close to the contact distance is trickier because positions that correspond to a perfect fitting between crystallographic planes of the two structures are extremely favorable, while the distances with some mismatch are hard to sample without the use of very strong force constant values.

The error bars in each *pmf* were estimated by the bootstrap method by dividing the data from the umbrella sampling simulations into five sets and computing the *pmf* from each data set, which are shown in Figures S8 and S9 in the Supporting Information file.

## Data Availability

A preprint version of this manuscript prior to the revisions is available: K.B. “How domain segregation in ionic liquids stabilizes nanoparticles and establish long-range ordering–a computational study”. 2024 10.26434/chemrxiv-2024–9mf09. ChemRxiv. URL: https://chemrxiv.org/engage/chemrxiv/article-details/660f1ef6418a5379b01762fb (accessed July 11, 2024).

## Supporting Information Available

Organization of the ionic liquid around a hydrophilic NP with increased interaction with the anion; NP–NP interaction energy in the dispersions; interaction parameters used in the simulations; initial arrangement of the NPs in the dispersions, and histograms of the position sampling across the umbrella sampling simulations and *pmf* replicas used to estimate the error bars (PDF)
