# Supplementary material for: How Domain Segregation in Ionic Liquids Stabilizes Nanoparticles and Establishes Long-Range Ordering—A Computational Study
Source: ACS Nano. 2024 Jul 27;18(31):20389–400. doi: 10.1021/acsnano.4c04581 (PMC11308924; doi:10.1021/acsnano.4c04581)
Supplement: Supplementary file 1 — nn4c04581_si_001.pdf [file nn4c04581_si_001.pdf]

Supporting Information for

How domain segregation in ionic liquids stabilizes nanoparticles and establish long-range ordering – a computational study

*Kalil Bernardino**

* kalilb@ufscar.br

Laboratório de Química Teórica, Departamento de Química, Universidade Federal de São Carlos, Rod. Washington Luiz S/n, 13565-905 São Carlos, Brazil

Contents:

1. Energy components for the NP-NP and NP-IL interaction at the pmf minimum and at maximum distance 1
2. Effect of the NP sites – anion interaction over the organization of the IL 2
3. Time evolution of the NP – NP interaction energy in the concentrated dispersions 3
4. Structures of solvent ions and of NPs and parameters attribution 5
5. Interaction parameters between NPs and between NPs and ILs 6
6. Histograms showing the sampling across the reaction coordinate of the pmfs 7
7. Individual pmfs obtained by bootstrap method in order to estimate error bars 9

Table S1 – Aggregation free energy and NP-NP, NP-cation* and NP-anion* interaction energy at the minimum of the potential of mean force (4.46 nm) and at the large separation (12.30 nm). Values in kJ/mol.

			Interaction energy at *r*=4.46 nm			Interaction energy at *r*=12.30 nm		
NP	solvent	$\Delta_{ag}G$	NP-NP	NP-cation	NP-anion	NP-NP	NP-cation	NP-anion
Hydrophilic	C4	-286±4	-306±6	-3170±57	-4136±86	0±0	-3346±58	-4379±87
Hydrophobic	C4	-227±6	-242±6	-3813±80	-1281±35	0±0	-4094±79	-1355±36
Hydrophilic	C8	-292±5	-305±6	-3041±55	-4441±114	0±0	-3208±60	-4727±122
Hydrophobic	C8	-137±6	-241±5	-5637±97	-257±23	0±0	-6013±97	-211±23

* Values for the interaction of the dimer. The interaction energy of a each NP with the ions is half of those figures.

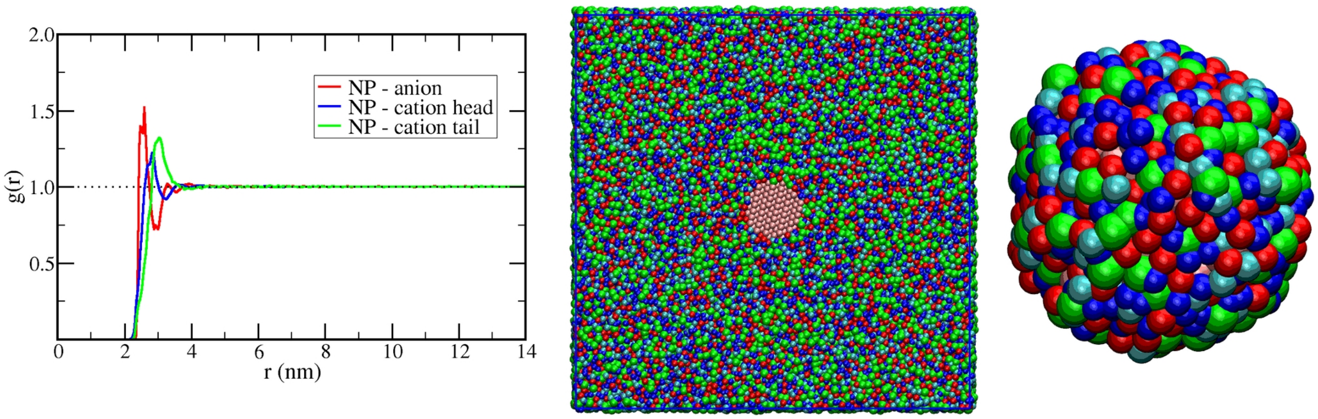

Figure S1 – Structure of the solvent around hydrophilic NP with the interaction parameter ε between NPs sites and the anion increased by 1.5 in the ionic liquid 1-butyl-3-methyl-imidazolium tetrafluoroborate (C4). Left: Radial distribution function of anion, cation imidazolium group and cation alkyl group around NP center of mass. Right: Graphical representations of the final structure showing a transversal slice of the simulation box with the NP in the center and the first shell (0.6 nm from any NP site) of interaction sites around the NP, with sites of the polar portion of the cation showed in blue (cyan for the uncharged site and dark blue for the charged ones), green for the hydrophobic portion of the cation and red for the anions.

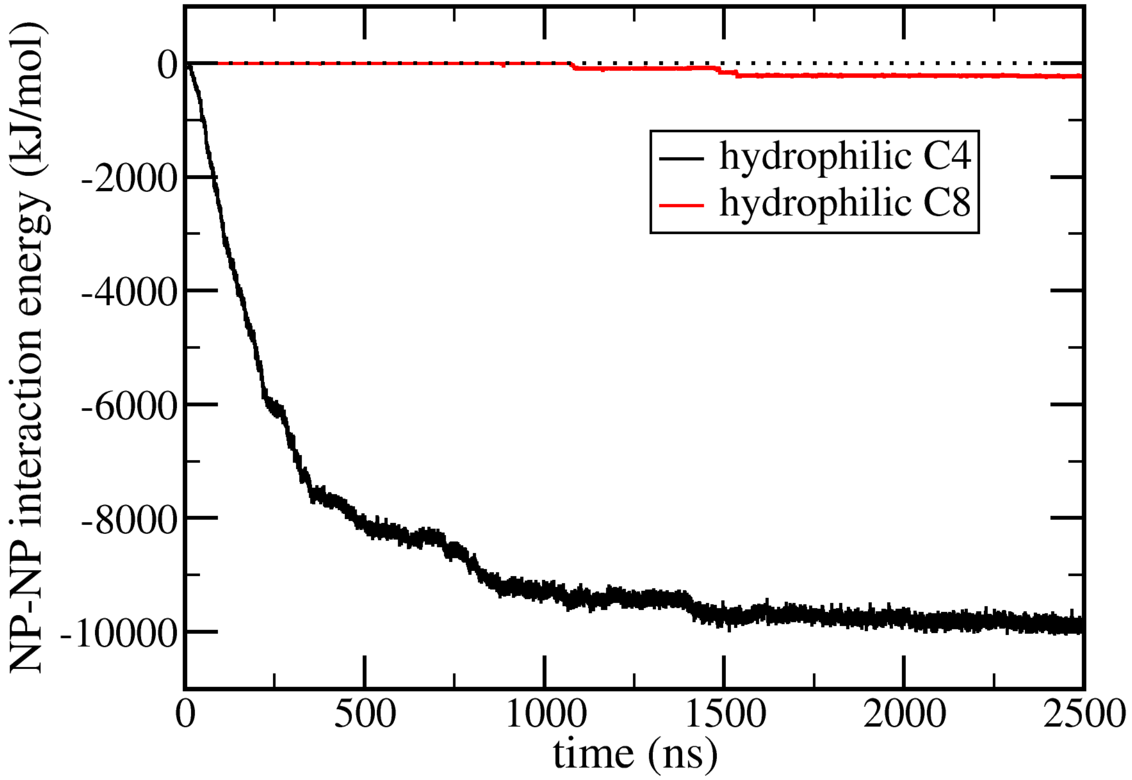

Figure S2 – Evolution of the contribution of the interaction between NPs to the system energy for the concentrated dispersions with hydrophilic NPs. Internal energy of the NPs were excluded from the analyses.

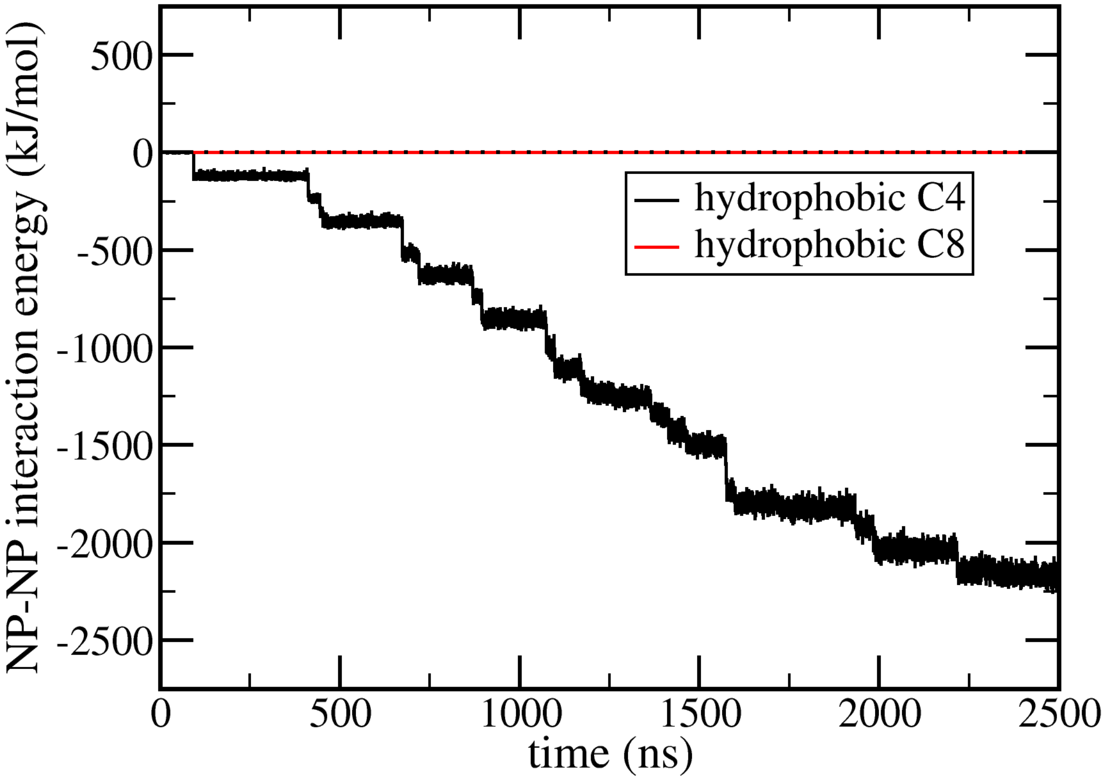

Figure S3 – Evolution of the contribution of the interaction between NPs to the system energy for the concentrated dispersions with hydrophobic NPs. Internal energy of the NPs were excluded from the analyses.

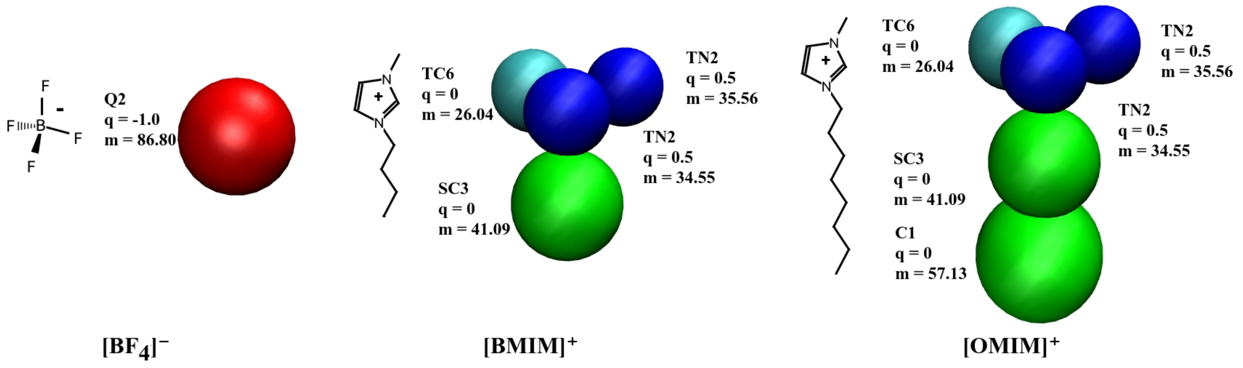

Figure S4 – Structural formulas and Martini 3.0 types, partial charges (in e) and masses (in g/mol) of ions used in this work.

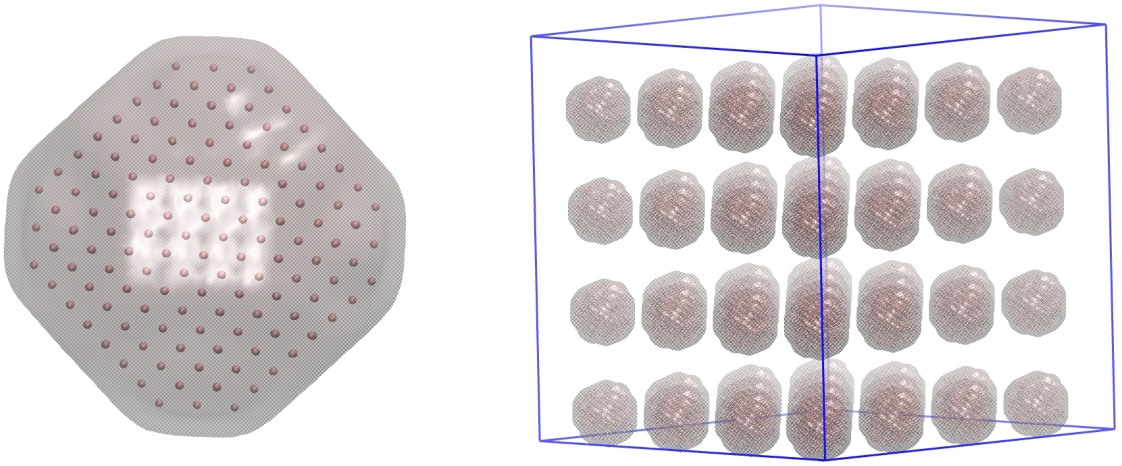

Figure S5 – Graphical representations of the structure of a single NP (left) and of the disposition of the 64 NPs in the initial structure for the simulations of the concentrated dispersions (right). Positions of interaction sites are represented by pink spheres while the NP surface is represented as a translucid solid surface.

Table S2 – Interaction parameters for the Lennard-Jones potential (Equation S1) between NPs interaction sites with sites from other NPs, IL cation and IL anion (see the Methods section and Figure 9 for details on type attribution).

Species	Type in Martini 3.0 notation	With hydrophilic NP (type P4)		With hydrophobic NP (type C1)	
		ε (kJ/mol)	σ (nm)	ε (kJ/mol)	σ (nm)
NP sites	P4 or C1	4.250	0.470	3.390	0.470
Cation head	TC6	1.850	0.395	1.670	0.395
	TN2	2.110	0.395	1.670	0.395
Cation tail	SC3	1.930	0.430	2.920	0.430
	C1	2.440	0.470	3.390	0.470
Anion	Q2	5.166	0.470	2.370	0.520

$$U_{LJ} = 4\varepsilon\left[\left(\frac{\sigma}{r}\right)^{12} - \left(\frac{\sigma}{r}\right)^{6}\right] \quad \text{(Equation S1)}$$

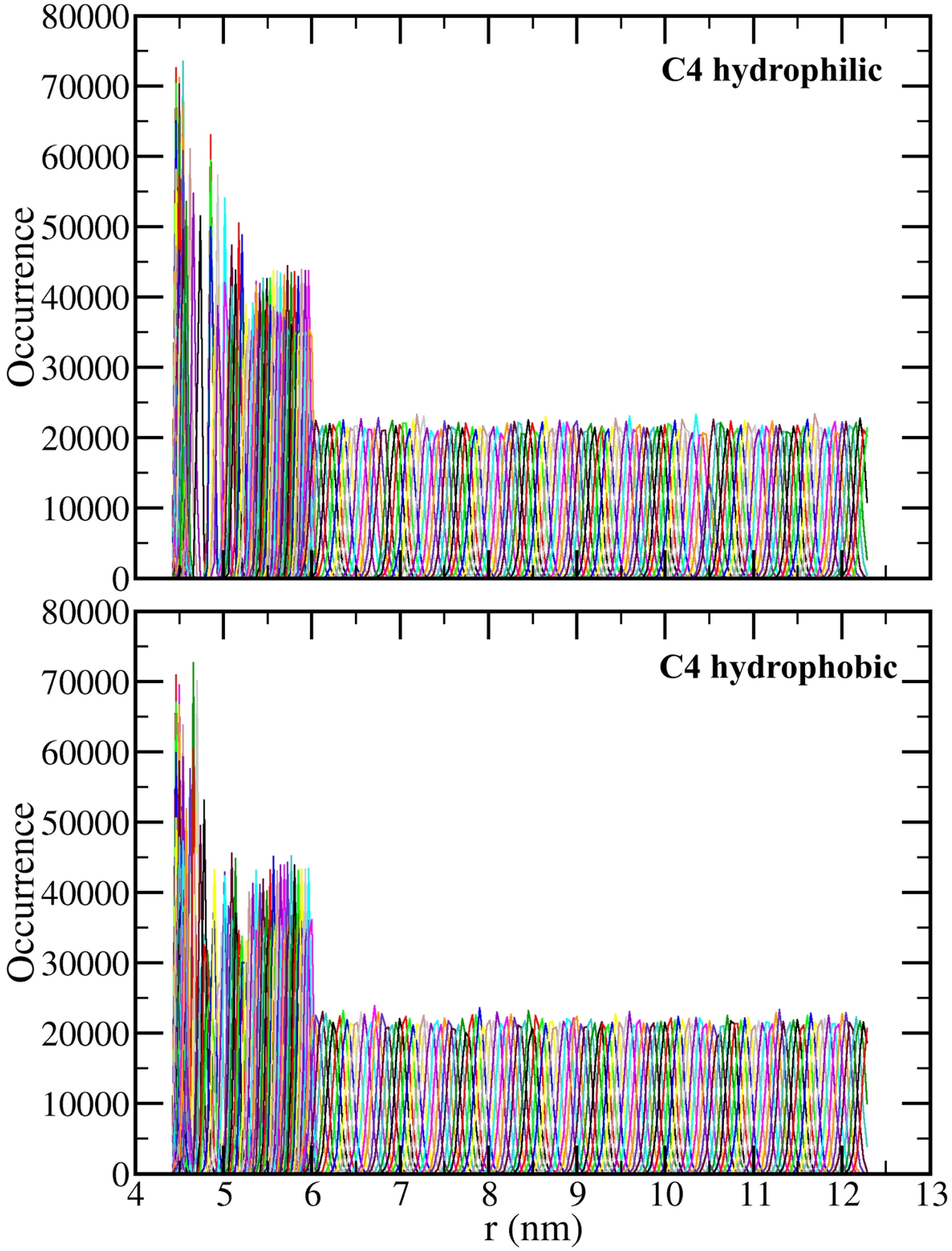

Figure S6 – Histograms showing the sampled values of the distance r in the pmf calculation between hydrophilic (top) and hydrophobic (bottom) NPs in [BMIM][BF4] showing that every distance between 4.4 and 12.3 were well-sampled. Each distribution corresponds to one simulation with the bias potential minimum in a different distance. Larger force constants and smaller separation between consecutive sampling windows were used for r ≤ 6.0.

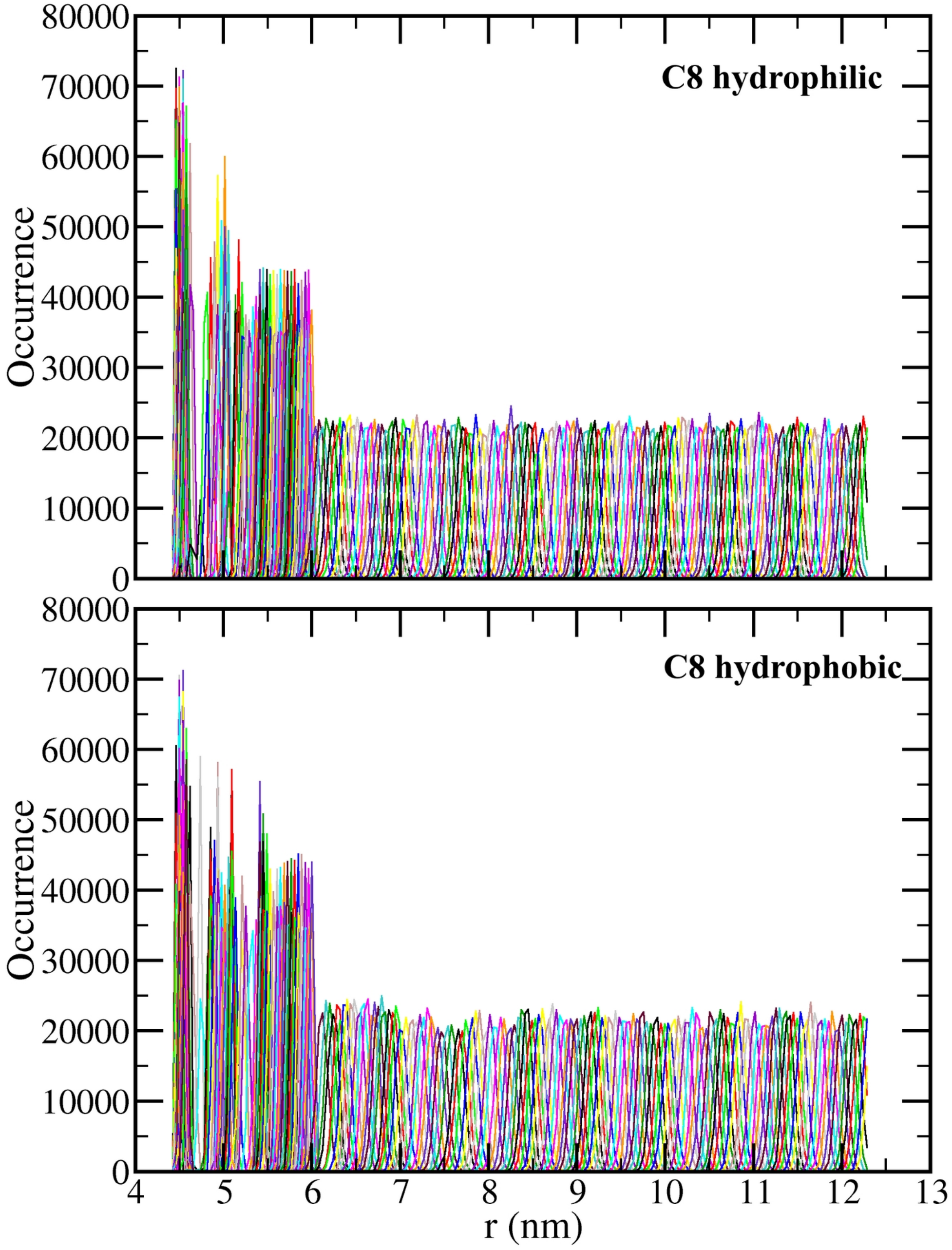

Figure S7 – Histograms showing the sampled values of the distance r in the pmf calculation between hydrophilic (top) and hydrophobic (bottom) NPs in [OMIM][BF4] showing that every distance between 4.4 and 12.3 were well-sampled. Each distribution corresponds to one simulation with the bias potential minimum in a different distance. Larger force constants and smaller separation between consecutive sampling windows were used for $r \leq 6.0$.

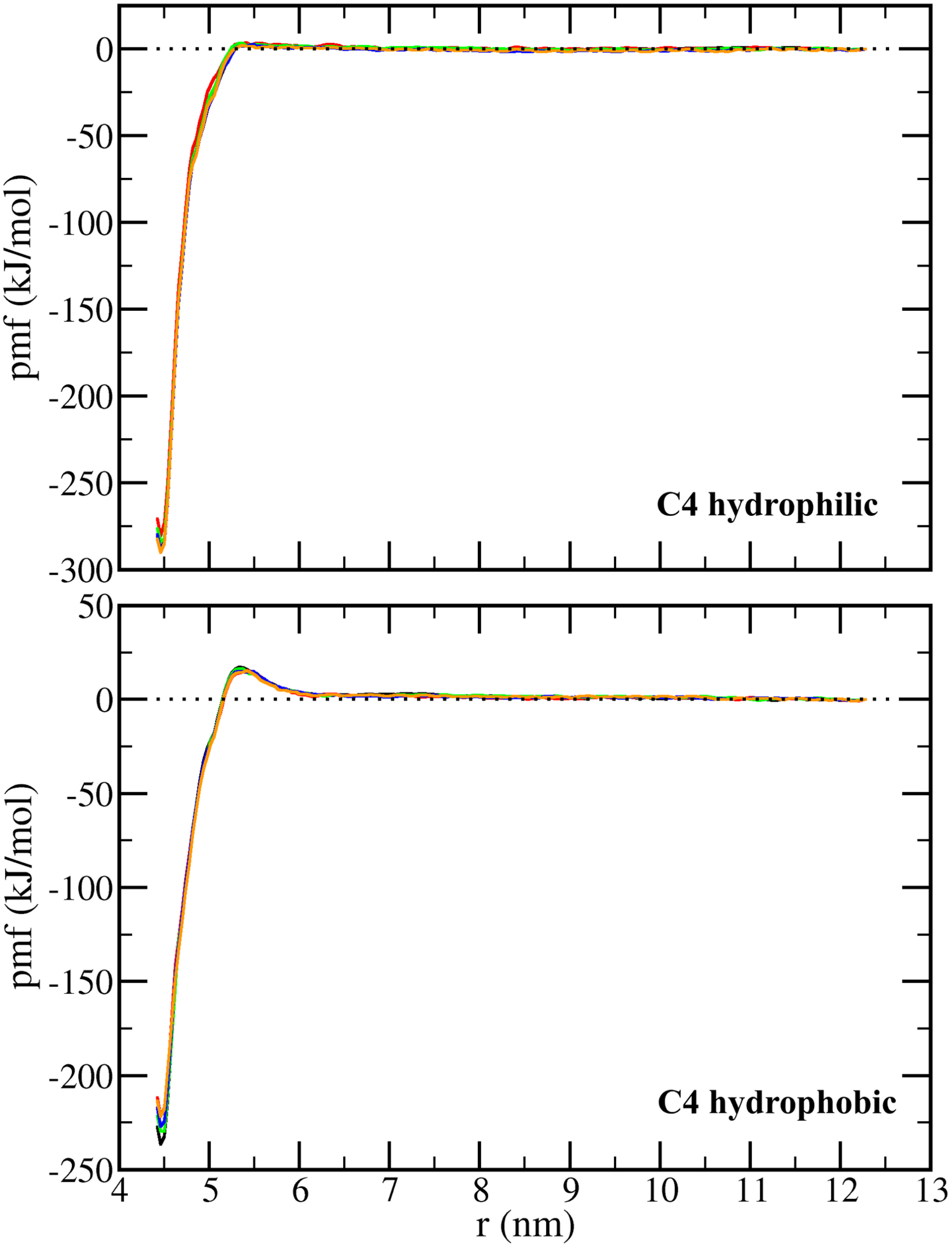

Figure S8 – Individual pmfs obtained by splitting the data used in the pmf calculation of hydrophilic (top) and hydrophobic (bottom) NPs in [BMIM][BF4] in five sets in order to estimate the error bars in Figure 1.

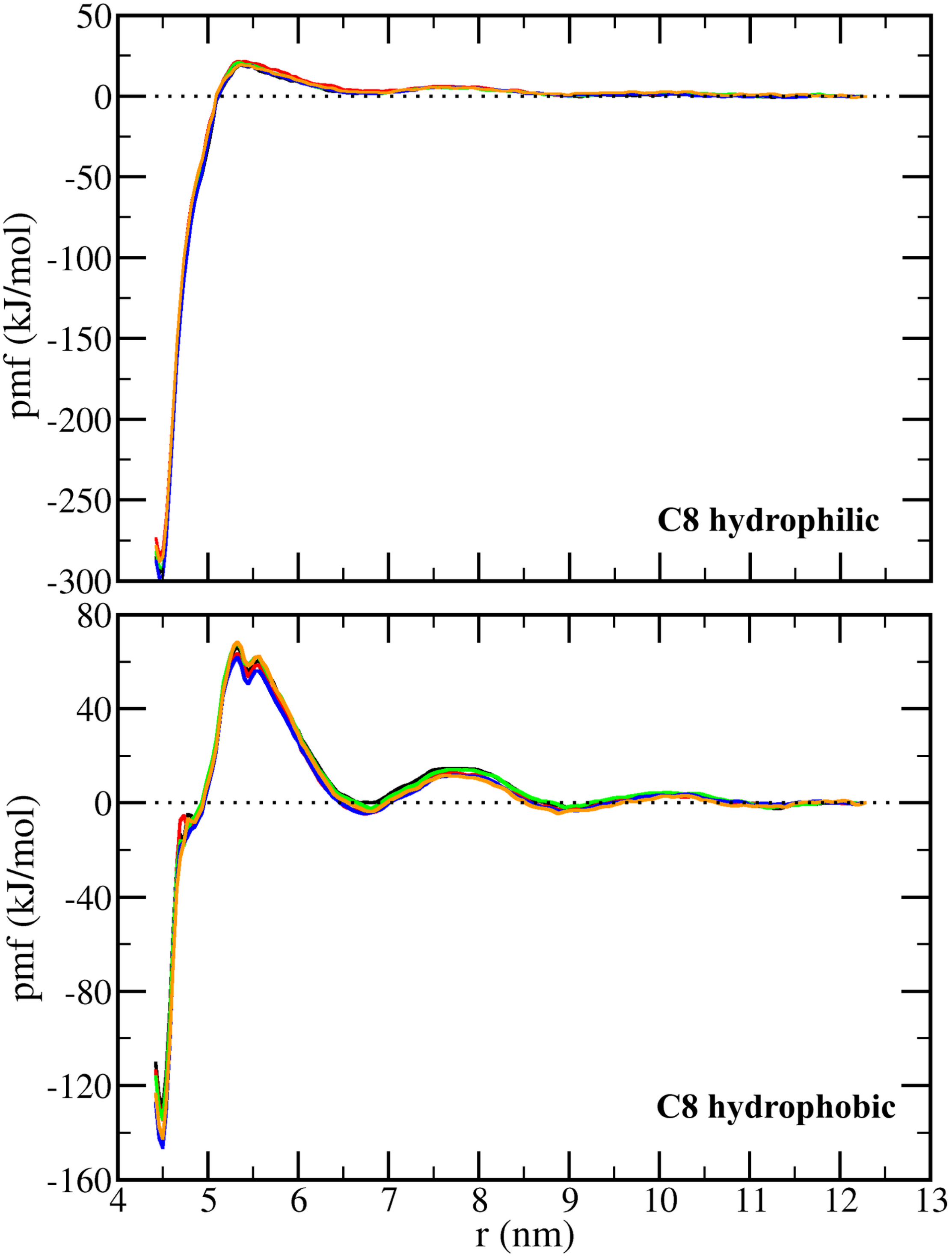

Figure S9 – Individual pmfs obtained by splitting the data used in the pmf calculation of hydrophilic (top) and hydrophobic (bottom) NPs in [OMIM][BF4] in five sets in order to estimate the error bars in Figure 1.
